# Supplementary material for: Global antimicrobial resistance and use surveillance system (GLASS 2022): Investigating the relationship between antimicrobial resistance and antimicrobial consumption data across the participating countries
Source: PLoS One. 2024 Feb 5;19(2):e0297921. doi: 10.1371/journal.pone.0297921 (PMC10843100; doi:10.1371/journal.pone.0297921)
Supplement: Attachment — Submitted filename: Response to reviewers_January 2024.docx [file pone.0297921.s001.docx]

PONE-D-23-32918

Global Antimicrobial Resistance and Use Surveillance System (GLASS 2022): Investigating the relationship between antimicrobial resistance and antimicrobial consumption data across the participating countries.

PLOS ONE

Dear Dr. Babafela,

Thank you for submitting your manuscript to PLOS ONE. After careful consideration, we feel that it has merit but does not fully meet PLOS ONE’s publication criteria as it currently stands. Therefore, we invite you to submit a revised version of the manuscript that addresses the points raised during the review process.

Please submit your revised manuscript by Jan 05 2024 11:59PM. If you will need more time than this to complete your revisions, please reply to this message or contact the journal office at [plosone@plos.org](mailto:plosone@plos.org). When you're ready to submit your revision, log on to [https://www.editorialmanager.com/pone/](https://nam04.safelinks.protection.outlook.com/?url=https%3A%2F%2Fwww.editorialmanager.com%2Fpone%2F&data=05%7C01%7Csajulo%40ttu.edu%7C8dfa94ecf9b246b75e4e08dbef5b9238%7C178a51bf8b2049ffb65556245d5c173c%7C0%7C0%7C638366948792299689%7CUnknown%7CTWFpbGZsb3d8eyJWIjoiMC4wLjAwMDAiLCJQIjoiV2luMzIiLCJBTiI6Ik1haWwiLCJXVCI6Mn0%3D%7C3000%7C%7C%7C&sdata=xwDkiUtRvBPTp7YXjFg5uydJrPlUDwEjx6je25BHTQg%3D&reserved=0) and select the 'Submissions Needing Revision' folder to locate your manuscript file.

Please include the following items when submitting your revised manuscript:

- A rebuttal letter that responds to each point raised by the academic editor and reviewer(s). You should upload this letter as a separate file labeled 'Response to Reviewers'.
- A marked-up copy of your manuscript that highlights changes made to the original version. You should upload this as a separate file labeled 'Revised Manuscript with Track Changes'.
- An unmarked version of your revised paper without tracked changes. You should upload this as a separate file labeled 'Manuscript'.

If you would like to make changes to your financial disclosure, please include your updated statement in your cover letter. Guidelines for resubmitting your figure files are available below the reviewer comments at the end of this letter.

If applicable, we recommend that you deposit your laboratory protocols in protocols.io to enhance the reproducibility of your results. Protocols.io assigns your protocol its own identifier (DOI) so that it can be cited independently in the future. For instructions see: [https://journals.plos.org/plosone/s/submission-guidelines#loc-laboratory-protocols](https://nam04.safelinks.protection.outlook.com/?url=https%3A%2F%2Fjournals.plos.org%2Fplosone%2Fs%2Fsubmission-guidelines%23loc-laboratory-protocols&data=05%7C01%7Csajulo%40ttu.edu%7C8dfa94ecf9b246b75e4e08dbef5b9238%7C178a51bf8b2049ffb65556245d5c173c%7C0%7C0%7C638366948792455988%7CUnknown%7CTWFpbGZsb3d8eyJWIjoiMC4wLjAwMDAiLCJQIjoiV2luMzIiLCJBTiI6Ik1haWwiLCJXVCI6Mn0%3D%7C3000%7C%7C%7C&sdata=24okZXLnpzteGXbWXmfx2D880ZqCIgq8h9yrJGQOBFM%3D&reserved=0). Additionally, PLOS ONE offers an option for publishing peer-reviewed Lab Protocol articles, which describe protocols hosted on protocols.io. Read more information on sharing protocols at [https://plos.org/protocols?utm_medium=editorial-email&utm_source=authorletters&utm_campaign=protocols](https://nam04.safelinks.protection.outlook.com/?url=https%3A%2F%2Fplos.org%2Fprotocols%3Futm_medium%3Deditorial-email%26utm_source%3Dauthorletters%26utm_campaign%3Dprotocols&data=05%7C01%7Csajulo%40ttu.edu%7C8dfa94ecf9b246b75e4e08dbef5b9238%7C178a51bf8b2049ffb65556245d5c173c%7C0%7C0%7C638366948792455988%7CUnknown%7CTWFpbGZsb3d8eyJWIjoiMC4wLjAwMDAiLCJQIjoiV2luMzIiLCJBTiI6Ik1haWwiLCJXVCI6Mn0%3D%7C3000%7C%7C%7C&sdata=c47b1Tg8BpzT6%2FriMZAVABC2NqspTtSRGDdNM4N8AbI%3D&reserved=0).

We look forward to receiving your revised manuscript.

Kind regards,

Balew Arega Negatie, Msc,MD
Academic Editor
PLOS ONE

Journal requirements:

1. When submitting your revision, we need you to address these additional requirements.

Please ensure that your manuscript meets PLOS ONE's style requirements, including those for file naming. The PLOS ONE style templates can be found at 
[https://journals.plos.org/plosone/s/file?id=wjVg/PLOSOne_formatting_sample_main_body.pdf](https://nam04.safelinks.protection.outlook.com/?url=https%3A%2F%2Fjournals.plos.org%2Fplosone%2Fs%2Ffile%3Fid%3DwjVg%2FPLOSOne_formatting_sample_main_body.pdf&data=05%7C01%7Csajulo%40ttu.edu%7C8dfa94ecf9b246b75e4e08dbef5b9238%7C178a51bf8b2049ffb65556245d5c173c%7C0%7C0%7C638366948792455988%7CUnknown%7CTWFpbGZsb3d8eyJWIjoiMC4wLjAwMDAiLCJQIjoiV2luMzIiLCJBTiI6Ik1haWwiLCJXVCI6Mn0%3D%7C3000%7C%7C%7C&sdata=Zu2nZLgmQU8JAxPnHOZeS21IY6hWBni7%2F3D0J1H5QuQ%3D&reserved=0) and 
[https://journals.plos.org/plosone/s/file?id=ba62/PLOSOne_formatting_sample_title_authors_affiliations.pdf](https://nam04.safelinks.protection.outlook.com/?url=https%3A%2F%2Fjournals.plos.org%2Fplosone%2Fs%2Ffile%3Fid%3Dba62%2FPLOSOne_formatting_sample_title_authors_affiliations.pdf&data=05%7C01%7Csajulo%40ttu.edu%7C8dfa94ecf9b246b75e4e08dbef5b9238%7C178a51bf8b2049ffb65556245d5c173c%7C0%7C0%7C638366948792455988%7CUnknown%7CTWFpbGZsb3d8eyJWIjoiMC4wLjAwMDAiLCJQIjoiV2luMzIiLCJBTiI6Ik1haWwiLCJXVCI6Mn0%3D%7C3000%7C%7C%7C&sdata=QFX3IqgjQtLn00Os7oPZYoFA4s9J6KFHILSHbnBbKV0%3D&reserved=0).

2. In your Data Availability statement, you have not specified where the minimal data set underlying the results described in your manuscript can be found. PLOS defines a study's minimal data set as the underlying data used to reach the conclusions drawn in the manuscript and any additional data required to replicate the reported study findings in their entirety. All PLOS journals require that the minimal data set be made fully available. For more information about our data policy, please see [http://journals.plos.org/plosone/s/data-availability](https://nam04.safelinks.protection.outlook.com/?url=http%3A%2F%2Fjournals.plos.org%2Fplosone%2Fs%2Fdata-availability&data=05%7C01%7Csajulo%40ttu.edu%7C8dfa94ecf9b246b75e4e08dbef5b9238%7C178a51bf8b2049ffb65556245d5c173c%7C0%7C0%7C638366948792455988%7CUnknown%7CTWFpbGZsb3d8eyJWIjoiMC4wLjAwMDAiLCJQIjoiV2luMzIiLCJBTiI6Ik1haWwiLCJXVCI6Mn0%3D%7C3000%7C%7C%7C&sdata=AoagQ0NzRft4p536ptWn%2FlyM1FmwXR30P8BrDomV%2FSI%3D&reserved=0).

""Upon re-submitting your revised manuscript, please upload your study’s minimal underlying data set as either Supporting Information files or to a stable, public repository and include the relevant URLs, DOIs, or accession numbers within your revised cover letter. For a list of acceptable repositories, please see [http://journals.plos.org/plosone/s/data-availability#loc-recommended-repositories](https://nam04.safelinks.protection.outlook.com/?url=http%3A%2F%2Fjournals.plos.org%2Fplosone%2Fs%2Fdata-availability%23loc-recommended-repositories&data=05%7C01%7Csajulo%40ttu.edu%7C8dfa94ecf9b246b75e4e08dbef5b9238%7C178a51bf8b2049ffb65556245d5c173c%7C0%7C0%7C638366948792455988%7CUnknown%7CTWFpbGZsb3d8eyJWIjoiMC4wLjAwMDAiLCJQIjoiV2luMzIiLCJBTiI6Ik1haWwiLCJXVCI6Mn0%3D%7C3000%7C%7C%7C&sdata=dfhBgua6R%2BIF09WAbixEihcOjjcWROnJkp%2BeBm4lYec%3D&reserved=0). Any potentially identifying patient information must be fully anonymized.

Important: If there are ethical or legal restrictions to sharing your data publicly, please explain these restrictions in detail. Please see our guidelines for more information on what we consider unacceptable restrictions to publicly sharing data: [http://journals.plos.org/plosone/s/data-availability#loc-unacceptable-data-access-restrictions](https://nam04.safelinks.protection.outlook.com/?url=http%3A%2F%2Fjournals.plos.org%2Fplosone%2Fs%2Fdata-availability%23loc-unacceptable-data-access-restrictions&data=05%7C01%7Csajulo%40ttu.edu%7C8dfa94ecf9b246b75e4e08dbef5b9238%7C178a51bf8b2049ffb65556245d5c173c%7C0%7C0%7C638366948792455988%7CUnknown%7CTWFpbGZsb3d8eyJWIjoiMC4wLjAwMDAiLCJQIjoiV2luMzIiLCJBTiI6Ik1haWwiLCJXVCI6Mn0%3D%7C3000%7C%7C%7C&sdata=z%2FmtA8cFgGBE3YPCLVQRctv5d6YiJJRV88bJvp59%2BuU%3D&reserved=0). Note that it is not acceptable for the authors to be the sole named individuals responsible for ensuring data access.

We will update your Data Availability statement to reflect the information you provide in your cover letter.

3. Please review your reference list to ensure that it is complete and correct. If you have cited papers that have been retracted, please include the rationale for doing so in the manuscript text, or remove these references and replace them with relevant current references. Any changes to the reference list should be mentioned in the rebuttal letter that accompanies your revised manuscript. If you need to cite a retracted article, indicate the article’s retracted status in the References list and also include a citation and full reference for the retraction notice.


Reviewers' comments:

Reviewer's Responses to Questions

**Comments to the Author**

1. Is the manuscript technically sound, and do the data support the conclusions?

The manuscript must describe a technically sound piece of scientific research with data that supports the conclusions. Experiments must have been conducted rigorously, with appropriate controls, replication, and sample sizes. The conclusions must be drawn appropriately based on the data presented.

Reviewer #1: Yes

Reviewer #2: Yes

2. Has the statistical analysis been performed appropriately and rigorously?

Reviewer #1: Yes

Reviewer #2: Yes

3. Have the authors made all data underlying the findings in their manuscript fully available?

The [PLOS Data policy](https://nam04.safelinks.protection.outlook.com/?url=http%3A%2F%2Fwww.plosone.org%2Fstatic%2Fpolicies.action%23sharing&data=05%7C01%7Csajulo%40ttu.edu%7C8dfa94ecf9b246b75e4e08dbef5b9238%7C178a51bf8b2049ffb65556245d5c173c%7C0%7C0%7C638366948792455988%7CUnknown%7CTWFpbGZsb3d8eyJWIjoiMC4wLjAwMDAiLCJQIjoiV2luMzIiLCJBTiI6Ik1haWwiLCJXVCI6Mn0%3D%7C3000%7C%7C%7C&sdata=cSoS%2B9MjH88Gb2hTOj0TfulD2Raf3kP3wELm6Nz5mjU%3D&reserved=0) requires authors to make all data underlying the findings described in their manuscript fully available without restriction, with rare exception (please refer to the Data Availability Statement in the manuscript PDF file). The data should be provided as part of the manuscript or its supporting information or deposited to a public repository. For example, in addition to summary statistics, the data points behind means, medians and variance measures should be available. If there are restrictions on publicly sharing data—e.g. participant privacy or use of data from a third party—those must be specified.

Reviewer #1: Yes

Reviewer #2: Yes

4. Is the manuscript presented in an intelligible fashion and written in standard English?

PLOS ONE does not copyedit accepted manuscripts, so the language in submitted articles must be clear, correct, and unambiguous. Any typographical or grammatical errors should be corrected at revision, so please note any specific errors here.

Reviewer #1: Yes

Reviewer #2: No

5. Review Comments to the Author

Please use the space provided to explain your answers to the questions above. You may also include additional comments for the author, including concerns about dual publication, research ethics, or publication ethics. (Please upload your review as an attachment if it exceeds 20,000 characters)

Reviewer #1: The manuscript is well written, and the findings will add to the body of evidence on association of antimicrobial resistance with antimicrobial consumption (AMC). However, drawing a direct correlation of the AMCs of participating countries with the resistance may not necessarily provide an accurate picture of the actual relationship owing to the varying data sources and the comprehensiveness thereof. This is evident from finding of country groupings into low consumption-low resistance, high-consumption high resistance, low consumption - high resistance and so forth. The contribution of the confounding factors may be elaborated under the study limitations.

As for the findings of statistically significant higher consumption of beta lactam penicillin and cephalosporins amongst the ATC groups, the GLASS report is self-explanatory even without application of any statistical method. Suggested to focus on the findings of correlation between resistance and consumption.

Abilities of the countries to implement antimicrobial stewardship interventions including infection control in the healthcare facilities would also have a huge bearing on the AMR and the paradox of LICs having high resistance even with low consumption could be because of lack of resources to implement this intervention. This may also be touched upon under the discussion.

**Response:** Thank you so much for pointing this out. We have been able to point out with appropriate references, that the lack of resources to implement infection control intervention is a factor for the observed paradox of high resistance and low consumptions in LMICs.

Line 188- 199: Is the word "recovery" appropriate in the context of 'odds of isolation/detection of resistant organism' reported by the countries? Suggested to replace with a suitable alternative.

**Response:** Thank you for your observations and comments to ensure the clarity of this manuscript. We have replaced “recovery” with isolation which appear appropriate in this situation.

Reviewer #2: This article provides crucial information to link the reported AMR data with the AMC data in different countries and regions based on the reported GLASS data, focusing on beta-lactam/cephalosporin and quinolones AMC data and beta-lactam/cephalosporin and quinolones resistant E. coli and Klebsiella pneumoniae and also explore the relationship and differences between the AMC data reported among the participating countries.
The authors performed critical analysis to gain better insight into GLASS data and makes inferences for integrated analysis of AMR and AMC data and to inform AMR intervention strategies at a global scale.

This study has great prospect to be useful to the international AMR community but needs to provide certain clarifications with minor revisions.

- The authors need to provide acknowledgements especially for the data source or any support received.

**Response:** Thank you for your observation and comment on this, we have now provided proper acknowledgment for the manuscript.

- The authors need to provide stronger rationale/ justification for limiting their analysis to blood stream infections and specifically E. coli and K. pneumonia, and also for the selected countries reported.

**Response:** Thank you for your comments. The reason for our focus on blood stream infections and specifically *E. coli* and *Klebsiella pneumoniae.* was partly outlined in lines 111-117, specifically because higher level of resistance associated with *klebsiella pneumoniae* globally and the worldwide spread of carbapenems-resistant Enterobacterales. We have further buttressed our rationale of limiting our analysis blood stream infections and specifically *E. coli* and *Klebsiella pneumoniae* because we are more likely to see a distinct more distinct relationship between antimicrobial use and resistance.

- Study limitations should be clearly articulated and separated from the conclusion session.

**Response:** Thank you for your observation and comments on this point. The limitations of this study have been separated from the conclusion. It has also been updated and rephrased for better clarity.

- The entire manuscript will benefit from English language editing for punctuations and use of words.

**Response:** We have worked on editing this manuscript to ensure clarity and proper punctuation.

- Intext referencing style needs to be adjusted and consistent.

**Response:** Thank you for your comment, appropriate changes have been made with the referencing

- Table 1: I assume that the reason for the bolding is to show significance, if so, then 0.0737 is not significant by your assumption of 0.05 significance level

**Response:** Thank you for your observation and comments on this point. We have adjusted the table to reflect these changes and we also made some changes in the result section lines 194-205 to reflect that only p value < 0.5 were considered in our analysis.

- The discussion session was adequate with robust deliberation on the study findings citing relevant literatures.
Minor revisions
-Abstract Line 33: State the actual p value, Line 37-38; DDD, ATC write in full at first appearance

**Response:** Thank you for our observations and comments. For our analysis the p value was set at < 0.5, This p value varied in the different analysis, however, only the results showing p value at < 0.5 were considered and this has been updated already in the manuscript. The DDD, ATC full meaning has also been updated in the abstract section.

- Pg 7 line 148: Add a full-stop after pneumonia

**Response:** Thank you for our observations and comments. We have updated this full-stop punctuation mark.

- Pg 10, line 227: “consistent with previous research…” please provide reference for this statement

**Response:** Thank you for our observations and comments. The studies that were referred to in the statement were cited immediately (line 225), citation number 36-38.

-Pg 12 line 258: “promoting the development of new antimicrobials…” Add a full stop before. …..This initiative

**Response:** Thank you for our observations and comments. We have updated this full-stop punctuation mark.

- Line 293 and 294: Add actual p value and not ≤0.05

**Response:** Thank you for our observations and comments. We added the reference to the table showing complete details about the individual p values. Since it is multiple comparison adding several different p-values that have been clearly outline in the result table might look clumsy to the readers, however, that was why we stated that p value < 0.05 as a way of summarizing the observations.

- Pg 13&14 Line 302-308: “Also the 2021……..” this sentence is too ling and unwinding and lacks clarity. Kindly separate into two sentences

**Response:** Thank you for our observations and comments. This has been clearly rewritten and separated into two sentences.

-Pg 16Line 355-357: This statement is not clear. Are you inferring that the reduced number of reporting sites was due to underreporting or underreporting was responsible for fewer sites in Iran. Either way, I fail to see the connection.

**Response:** Thank you for our observations and comments. This statement has been reviewed and edited to clearly explain the connection. The connection is that although Iran had few surveillance sites, other independent studies within the countries have confirmed high resistance rates, attributing it to misuse of antimicrobials.

-Pg 17 Line 388: Remove “application of “ to read as “such that beta-lactam…..” or rephrase the entire sentence for clarity

**Response:** Thank you for our observations and comments. This statement has been reviewed and paraphrased to ensure clarity.

-Line 411: Add a full stop before “therefore”

**Response:** Thank you for our observations and comments. We have updated this full-stop punctuation mark.

6. PLOS authors have the option to publish the peer review history of their article ([what does this mean?](https://nam04.safelinks.protection.outlook.com/?url=https%3A%2F%2Fjournals.plos.org%2Fplosone%2Fs%2Feditorial-and-peer-review-process%23loc-peer-review-history&data=05%7C01%7Csajulo%40ttu.edu%7C8dfa94ecf9b246b75e4e08dbef5b9238%7C178a51bf8b2049ffb65556245d5c173c%7C0%7C0%7C638366948792455988%7CUnknown%7CTWFpbGZsb3d8eyJWIjoiMC4wLjAwMDAiLCJQIjoiV2luMzIiLCJBTiI6Ik1haWwiLCJXVCI6Mn0%3D%7C3000%7C%7C%7C&sdata=4Qgf7Jfq17Tp00pZkz73Vnuakf3WHka9%2BcfbJlZSZWY%3D&reserved=0)). If published, this will include your full peer review and any attached files.

If you choose “no”, your identity will remain anonymous but your review may still be made public.

**Do you want your identity to be public for this peer review?** For information about this choice, including consent withdrawal, please see our [Privacy Policy](https://nam04.safelinks.protection.outlook.com/?url=https%3A%2F%2Fwww.plos.org%2Fprivacy-policy&data=05%7C01%7Csajulo%40ttu.edu%7C8dfa94ecf9b246b75e4e08dbef5b9238%7C178a51bf8b2049ffb65556245d5c173c%7C0%7C0%7C638366948792455988%7CUnknown%7CTWFpbGZsb3d8eyJWIjoiMC4wLjAwMDAiLCJQIjoiV2luMzIiLCJBTiI6Ik1haWwiLCJXVCI6Mn0%3D%7C3000%7C%7C%7C&sdata=kWEehghTkEF9D9RzhH%2BqwTK77UL0ZpvqgptNTRfb1d8%3D&reserved=0).

Reviewer #1: No

Reviewer #2: **Yes:** Emelda Eberechukwu Chukwu

[NOTE: If reviewer comments were submitted as an attachment file, they will be attached to this email and accessible via the submission site. Please log into your account, locate the manuscript record, and check for the action link "View Attachments". If this link does not appear, there are no attachment files.]

While revising your submission, please upload your figure files to the Preflight Analysis and Conversion Engine (PACE) digital diagnostic tool, [https://pacev2.apexcovantage.com/](https://nam04.safelinks.protection.outlook.com/?url=https%3A%2F%2Fpacev2.apexcovantage.com%2F&data=05%7C01%7Csajulo%40ttu.edu%7C8dfa94ecf9b246b75e4e08dbef5b9238%7C178a51bf8b2049ffb65556245d5c173c%7C0%7C0%7C638366948792455988%7CUnknown%7CTWFpbGZsb3d8eyJWIjoiMC4wLjAwMDAiLCJQIjoiV2luMzIiLCJBTiI6Ik1haWwiLCJXVCI6Mn0%3D%7C3000%7C%7C%7C&sdata=3JS6vmLTAI%2FIcHACPIWr75txj%2B9DIC2wuvsxljD9I6o%3D&reserved=0). PACE helps ensure that figures meet PLOS requirements. To use PACE, you must first register as a user. Registration is free. Then, login and navigate to the UPLOAD tab, where you will find detailed instructions on how to use the tool. If you encounter any issues or have any questions when using PACE, please email PLOS at [figures@plos.org](mailto:figures@plos.org). Please note that Supporting Information files do not need this step.

*In compliance with data protection regulations, you may request that we remove your personal registration details at any time.* [*(Remove my information/details)*](https://nam04.safelinks.protection.outlook.com/?url=https%3A%2F%2Fwww.editorialmanager.com%2Fpone%2Flogin.asp%3Fa%3Dr&data=05%7C01%7Csajulo%40ttu.edu%7C8dfa94ecf9b246b75e4e08dbef5b9238%7C178a51bf8b2049ffb65556245d5c173c%7C0%7C0%7C638366948792455988%7CUnknown%7CTWFpbGZsb3d8eyJWIjoiMC4wLjAwMDAiLCJQIjoiV2luMzIiLCJBTiI6Ik1haWwiLCJXVCI6Mn0%3D%7C3000%7C%7C%7C&sdata=JfPjaxNoADm%2BDWoZFM5nwogVul9A72hXCs9Y0QicWUM%3D&reserved=0)*. Please contact the publication office if you have any questions.*
